# Understanding the influence of social media on COVID-19 vaccine acceptance in a war-torn Syria: A cross-sectional study

**DOI:** 10.1097/MD.0000000000038956

**Published:** 2024-08-09

**Authors:** Areej Kahwaji, Thurya Alaryan, Hani Alhelwani, Moath Salem, Tamim Alsuliman

**Affiliations:** a Faculty of Pharmacy, Damascus University, Damascus, Syria; b Department of Pharmaceutics and Pharmaceutical Technology, Faculty of Pharmacy, Damascus University, Damascus, Syria; c Hôpital CHU Brugmann, Brussels, Belgium; d Faculty of Medicine, Damascus University, Damascus, Syria; e Sorbonne University, Paris, France; f Service d’d’Hématologie Clinique et Thérapie Cellulaire, Hôpital Saint-Antoine, AP-HP, Paris, France; g INSERM, Paris, France.

**Keywords:** COVID-19, infodemic, low-income country, social media, vaccine acceptance

## Abstract

Social media has become a source of disseminating information to the public during the COVID-19 outbreak which has been a great advantage for healthcare centers. However, foreign anti-vaccination campaigns on social media increased the disbelief in vaccine safety. To our knowledge, the effects of social media on COVID-19 vaccine acceptance are not well-studied in low-income countries. The primary objective of this survey is to investigate Syrians’ dependence on social media platforms to get information about vaccines, and to what extent it affects their vaccination decision. A web-based cross-sectional study was conducted in Syria from May 26th to July 26th, 2022 using an online questionnaire as Google Form posted on various social media platforms. The questionnaire consisted of 53 questions related to each of the socio-demographic characteristics, beliefs, and knowledge about COVID-19 vaccination, willingness to get vaccinated, and social media frequency use and its effects. Univariate and multivariate logistic regression was performed to identify factors associated with vaccination behavior. A total of 780 questionnaires were completed; around 42.2% of study participants did not get the vaccine, and 24% would take it only under compulsory rules. Also, only 3.08% of the participants answered correctly on the knowledge-evaluation questions. Results of the univariate analysis showed that being female, living in an urban residential area, and having good vaccine knowledge were positive predictors of vaccine receiving. The unvaccinated group had a higher likelihood of being college students, not trusting in the vaccine, knowing relatively less about the vaccine, and not having previously been exposed to the virus. No significant correlation between vaccination status and using social media was shown in our investigation. However, our results show the importance of social media information in health-related decisions in war-torn countries and emphasize further investigations to confirm causality and determine the best health policy choice.

## 1. Introduction

Social media has become a crucial source of information dissemination during the coronavirus outbreak due to its ability to quickly reach large audiences. Individuals utilized these platforms for various purposes, including seeking medical advice, gathering information about infection symptoms, and assessing their own health conditions.^[1]^ Meanwhile, healthcare organizations such as the Centers for Disease Control and Prevention (CDC), the World Health Organization (WHO), and other medical journals leveraged social media to regularly post guidance and share informative content through daily posts, podcasts, and YouTube videos across multiple platforms.^[2]^

These platforms also served as communication hubs for stakeholders during the pandemic. Government health authorities used their social media accounts as official sources to provide timely updates to local agencies and journalists.^[3]^ Additionally, many Arabic-speaking scientific initiatives used social media to raise local medical and scientific awareness. By publishing COVID-19-related articles, infographics, and diagrams, they spread evidence-based information about vaccines.^[4]^ Furthermore, communication applications played a significant role during quarantine, believed to mitigate the psychological impact of isolation by alleviating anxiety, boredom, and long-term distress.^[5]^

However, despite the benefits of social media, the spread of misinformation about the coronavirus poses a significant threat to public health. Misinformation can exacerbate health issues and propagate misleading conspiracy theories about COVID-19 vaccines.^[6]^ Although misinformation is a common issue nowadays, the effects of social media on COVID-19 vaccine acceptance are not well-studied in low-income countries. This study aims to investigate Syrians’ dependence on social media platforms to get information about vaccines and the impact of misleading information on the vaccination decisions of different levels of education individuals. Additionally, it aims to uncover the most common conspiracy theories circulating on social media platforms.

Vaccination reluctance is a long-standing phenomenon that is mainly led by anti-vaccine groups who distrust the information provided by health professionals and official sources about vaccines. These groups often turn to social networks that reinforce their doubts and beliefs. Social media campaigns by foreign anti-vaccine entities have further intensified skepticism about vaccine safety.^[7,8]^ A systematic review highlighted the use of Twitter (previously known as X) by anti-vaccine groups to spread false information, share personal stories about alleged side effects, promote claims about vaccine harms, discuss pharmaceutical profits, and circulate conspiracy theories with no evidence.^[8]^

In Ukraine, a study identified 5 anti-vaccine campaigns on social media that propagated negative messages about immunization, raising concerns about vaccine hesitancy and undermining confidence in official information sources about COVID-19.^[9]^ African countries are particularly vulnerable due to the widespread belief that Africa is less at risk of COVID-19. A study found significant dissemination of online misinformation during the pandemic, which could seriously threaten vaccine acceptance in regions where accurate information is scarce.^[10]^ In Nigeria, exposure to fake news led to greater negative perceptions of the COVID-19 vaccine, which did not significantly change even after counseling interventions.^[11]^

Meanwhile, in Syria, the situation is compounded by the devastating effects of years of violence on its public health sector making it even more challenging to implement a successful vaccination program.^[12–15]^ To the date of writing, only 18.3% of the population received at least their first dose of the vaccine that was provided by the COVAX international initiative.^[16]^ A nationwide study showed that only 37% of Syrians are willing to be vaccinated, while 31% were still hesitant.^[17]^ The challenges above among others make it difficult to timely implementation of vaccination putting pressure on an already fragile health system.

## 2. Methods

### 2.1. Study design and data collection

This cross-sectional study collected data from May 26th to July 26th, 2022 using an online questionnaire developed via Google Forms. The questionnaire was posted on various social media platforms, including Facebook, WhatsApp, and Telegram. We employed convenience sampling, distributing the questionnaire across diverse social media groups. These groups included a large number of members from different socio-economic backgrounds and age groups, and they covered major cities across the country, representing a broad spectrum of Syrian society. This method was deemed the most suitable for data collection given the safety concerns in some areas of Syria due to the war, which made in-person data collection impractical. During the distribution period, the number of daily confirmed COVID-19 cases in Syria remained stable or slightly rising.^[18]^ Vaccines were allegedly available at health centers and temporary COVID-19 vaccination sites. Nevertheless, vaccination was not mandatory in almost all settings and sectors, indicating that responders were free to choose to get vaccinated. Inclusion criteria were residing in Syria, being 18 years old or older, and being willing to take the questionnaire.

The sample size was determined using Steven K. Thompson sampling. A statistical power analysis was performed to calculate the sample size based on the number of COVID-19 vaccines provided to the Syrian Arab Republic via the COVAX international initiative. Electronic informed consent was obtained from research participants. The questionnaire was inspired by previous studies.^[19,20]^ Almost all participants (97%) agreed to have their data collected and used for research purposes. Disagreements were excluded.

### 2.2. Questionnaire

The self-structured questionnaire consisted of 53 questions divided into 3 sections; each with a specific subject:

Socio-demographic characteristics: This section includes 9 questions about gender, age group, marital status, educational level, financial status, working in a healthcare-related field, living with someone over 60, place of residence (city, suburb, or village), and area of residence.

*B*eliefs and knowledge about COVID-19 vaccination and willingness to get vaccinated: This section consisted of 27 questions that covered 5 topics. The first is vaccination willingness (2 questions). The second is previous infection with SARS-CoV-2 and the associated symptoms, along with the diagnosis method (5 questions). Thirdly, concomitant diseases and prescribed drugs (4 questions). After that, questions about smoking status (1 question), vaccination and non-vaccination motives (3 questions), and knowledge and perceptions toward COVID-19 vaccines (12 questions) followed respectively.

*Social Media frequency use and its effects:* 17 questions that investigate participants’ involvement on different social media platforms regarding each of COVID-19 vaccines, vaccine-related information publishing, reliability and confidence in sources of information published, information disseminated on social networks and its impact on vaccine receptivity, conspiracy theories regarding COVID-19 vaccines that spread through Arabic-speaking social networks, and participants’ perspectives concerning vaccination actions that officials should take in the future pandemics (full questionnaire, Supplemental Digital Content, http://links.lww.com/MD/N358).

### 2.3. Ethical approval and data availability

Ethical approval was obtained from the Medical Research Ethics Committee, Faculty of Pharmacy, Damascus University, Damascus, Syria on August 09, 2022 (Reference number 4064). Informed consent was collected from all participants prior to data collection.

In compliance with local laws and regulations, data was not shared publicly. However, the anonymized datasets used and/or analyzed during the current study are available from the corresponding author on reasonable request.

### 2.4. Data analysis

The investigators developed the self-structured questionnaire using a Google Forms online survey. Data gathered from the online survey was directly exported to an Excel spreadsheet. The raw data in the Excel sheet was then totally checked, de-identified, and encoded to comply with the statistics software.

Following that, IBM Statistical Package for Social Sciences version 26.0 (SPSS Inc., Chicago, IL) was used to perform the Pearson Chi-square test to investigate categorical associations, and ANOVA was applied to determine any statistically significant difference between 2 or more categorical groups. In addition, multivariate regression analysis was done to measure the linearity between variables. Frequencies and percentages were used to report categorical variables. Equally important, statistics were regarded as significant at *P* value < .05.

## 3. Results

### 3.1. Participants characteristics

Of 801 responses, 780 (a 97.4% response rate) agreed to participate in the research and completed the questionnaire. Approximately, half of the sample (52.4%) worked in a field other than the health-related field. 439 participants (56.3%) were single with the majority living in a home with residents under 60 years old. Regarding educational level, 416 (53.3%) were bachelor degree holders. Additionally, 442 (56.7%) reported that their expenditure covers only the basic needs of life. whereas, 338 (43.3%) had their basic needs along with complementary expenditures. All other subjects’ characteristics are demonstrated in Table 1.

**Table 1:** Representation of socio-demographic characteristics in the study population (N = 780).

Category	Factor	Frequency	Percent
Gender	Male	283	36.3%
	Female	488	62.6%
	Rather not say	9	1.1%
Age group	18–25	286	36.7%
	26–40	356	45.6%
	41–60	124	15.9%
	>60	14	1.8%
Living location	Northern governorates	91	11.7%
	Eastern governorates	16	2%
	Central governorates	95	12.2%
	Western governorates	88	11.3%
	Southern governorates	490	62.8%
Marital status	Single	439	56.3%
	Married	319	40.9%
	Divorced/Widowed	22	2.8%
Working in a healthcare-related field	Yes	371	47.6%
	No	409	52.4%
Having someone older than 65 yr at home	Yes	292	37.4%
	No	488	62.6%
Education level	No formal education	3	0.4%
	Middle school	28	3.6%
	Highschool	55	7%
	University student	194	24.9%
	Bachelor degree	416	53.3%
	Post-graduate degree	84	10.8%
Area of residency	City	582	67.7%
	Suburb	111	14.2%
	Village	141	18.1%
Financial Status	Basics of living need only	338	43.3%
	Basics of living needs along with complementary expenditure	442	56.7%

### 3.2. Beliefs and knowledge about COVID-19 vaccination and willingness to get vaccinated

62.4% of the participants declared to have had SARS-CoV-2 infection before with 63.1% stating to have contracted the virus at least once, and 28.3%, 7.2%, 1.4% to have contracted it twice, 3 times or more respectively. “Having the common clinical symptoms of COVID-19” was the leading diagnosis method to confirm the infection for nearly half of the participants (55.9%). Regarding symptom severity, 18.5% were asymptomatic or with minor symptoms only, and 36.9%, 14.4%, and 0.6% had either moderate, or severe without referring to a health center, or severe with symptoms requiring hospital admission, respectively. Interestingly, 657 (84.2%) declared no comorbidities, and 509 (65.3%) were nonsmokers.

**Figure 1.**
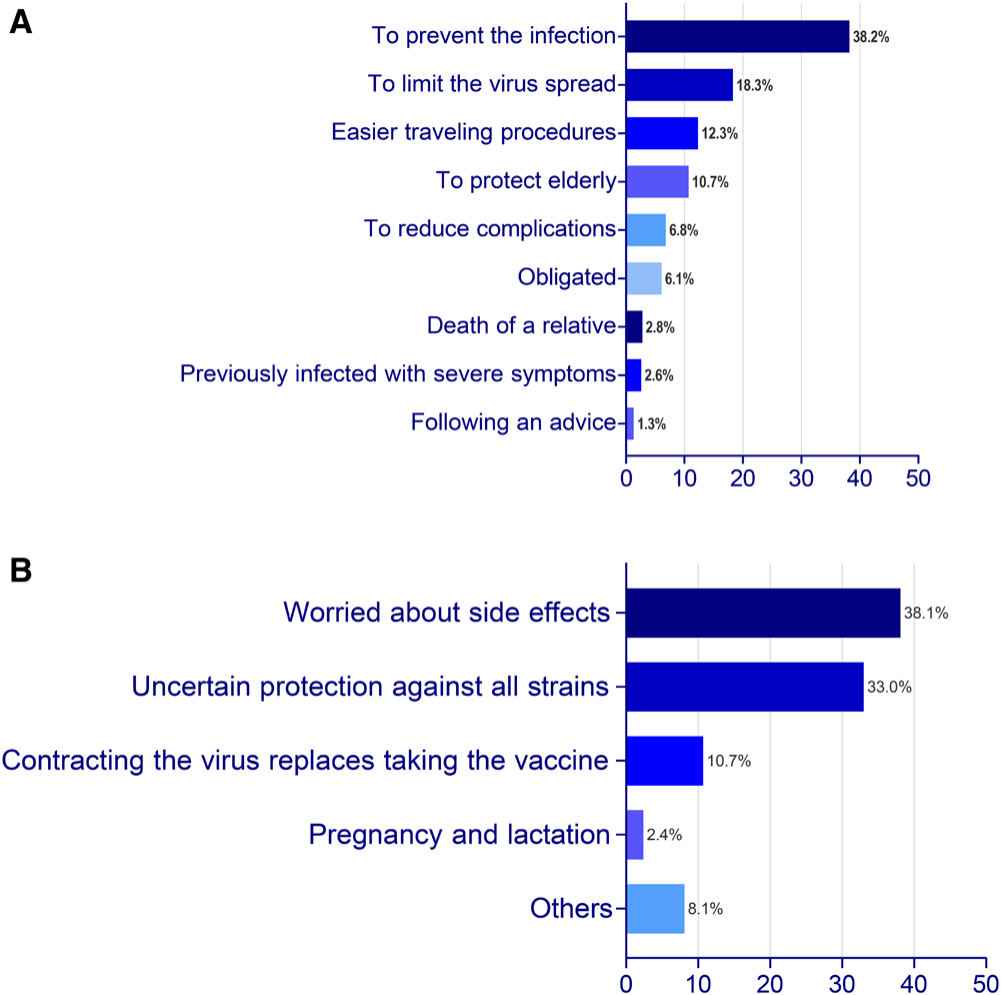
(A) Illustrates the motivations behind participants’ decision to receive the vaccine. (B) Illustrates the rationale behind participants’ decision not to receive the vaccine.

329 (42.2%) of study participants did not get the vaccine, of which 200 (60.8%) refrained from getting vaccinated in the future, and 24% accepted to take it only under compulsory rules. Furthermore, 39.2% received 2 doses of the vaccine and were considered fully vaccinated, while 14% received the one-dose vaccine, and 4.6% were partially vaccinated. In both groups, the reasons behind their vaccination decisions are shown in Figure 1. “Getting immunized against the virus and preventing future infections” was the leading cause (n = 175, 38.2%) in the vaccinated group, whereas “Fearing potential side effects” was the main drive (n = 128, 38.1%) for non-vaccination. Furthermore, around 490 (62.8%) of the participants believed that the vaccine reduces the severity of the infection or the duration of recovery.

Knowledge-evaluation regarding COVID-19 vaccines is represented in Table 2.

**Table 2:** Participants’ knowledge score about the COVID-19 vaccine.

Number of questions that were answered correctly	Frequency	Percent
0	2	0.25
1	15	1.92
2	27	3.46
3	40	5.13
4	53	6.79
5	70	8.97
6	103	13.33
7	109	14
8	120	15.38
9	127	16.28
10	89	11.41
11	24	3.08

### 3.3. Social media frequency use and vaccination status

**Figure 2.**
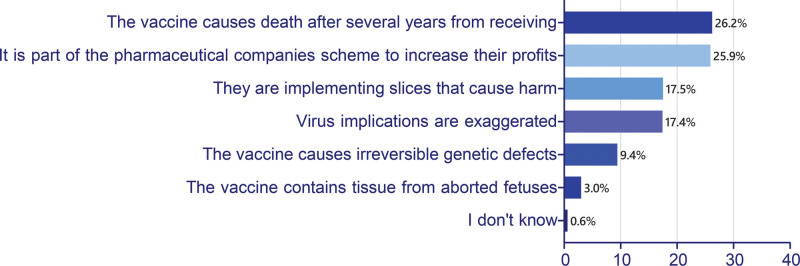
Conspiracy theories related to the COVID-19 vaccine propagated on Arabic-speaking social media platforms.

**Figure 3.**
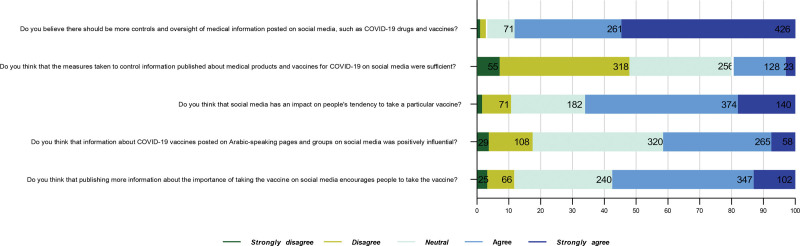
Participants’ attitudes toward social media and their opinions on regulatory measures.

Replying to “How many hours do you spend daily on social media platforms?” 335 (43%) of the participants spent between (2–4) hours while 31% spent more than 4 hours and 203 (26%) spent only between (1–2) hours daily. Regarding information sources about COVID-19 vaccines, 209 (26.8%) claimed to get vaccine information from health organizations’ websites, whereas 20.9% used social media to learn about vaccines. Facebook was used the most (48.7%). When asked “To what extent do you ‘rely on’ or ‘trust’ the content posted through various platforms?” more than half of the participants stated to trust (59.7%) or moderately rely on (52.3%) the posts’ information. Moreover, social media posts about vaccination importance had a positive encouragement on 44.5% of the participants. On the other hand, conspiracy theories on social media were considered the main reason to refuse the vaccines by 33.8%. The most read conspiracy “Vaccine causes death after several years from receiving,” signaled by 26.2%, and the second followed by 25.9% claimed, “The pandemic is part of the pharmaceutical manufacturing companies’ deceiving scheme to increase their profits after imposing vaccination.” Other conspiracy theories disseminated on Arabic-speaking social platforms are shown in Figure 2. Over 28.5% of the participants agreed that “implementing public awareness campaigns” is the most encouraging method to raise awareness of vaccination in future pandemics. This was followed closely by 25.5% who supported the approach of “sharing vaccinating reels of public figures and celebrities.” Figure 3 displays participants’ attitudes toward social media and their opinions on measures for its regulation.

### 3.4. Correlations between vaccination status and other variables

**Figure 4.**
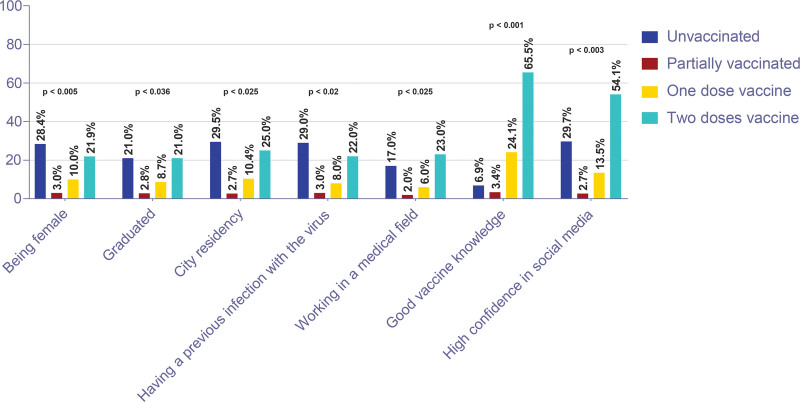
Variables demonstrating statistical significance (*P* value < .05) in the chi-square univariate analysis.

The results of the Chi-square univariate analysis; shown in Figure 4 revealed a statistically significant difference between vaccination status and other variables. Specifically, 28.4% of the female participants were still unvaccinated in comparison with 13.5% of males (*P* value < .005). Moreover, 29% of the participants who had been infected with the virus before were still unvaccinated (*P* value < .02). Furthermore, the majority of the unvaccinated group (29.5%, *P* value < .025) reside in the city areas. On the other hand, of the fully vaccinated group, nearly a fourth (23%) appeared to work in a medical-related field (*P* value < .025).

### 3.5. Multinomial regression analysis

The unvaccinated group was more likely to be either college students, not trusting in the vaccine, knowing relatively less about the vaccine, or have never been exposed to the virus (*P* < .001, *P* < .001, *P* < .03, and *P* < .019, respectively). On the other hand, city residents, college students, and graduates were more likely to receive the single-dose vaccine (*P* < .007, *P* < .048, and *P* < .016, respectively).

After vaccination, participants who followed prevention procedures tended to get vaccine information from medical sources, not social media. This group had a much lower infection rate than those who ignored safety precautions.

## 4. Discussion

The anti-vaccine movement is ever-growing on social media, with millions of followers spreading misinformation that could hinder any potential COVID-19 vaccine distribution in the future.^[21]^ Part of the responsibility could be attributed to the health authorities’ inadequate response facing COVID-19 infodemics. The inadequate response provided, to some extent, anti-vaccine groups with the best opportunity to successfully spread false information to the public. This chance was, in particular, at a crucial stage in the pandemic when vaccination access was improving and vaccination was urgently needed.^[22]^

To the best of our knowledge, this is the first study to examine the impact of social media on Syria public acceptance of the COVID-19 vaccine. The reliance on social media as a source of COVID-19 information imposes a major concern in the Arabophone world and has been linked to greater rates of vaccine hesitancy.^[19,23]^ Almost 42.2% of study participants were still unvaccinated, which is approximately half the national unvaccinated rate of 81.7% cited by the Johns Hopkins Coronavirus Resource Center.^[16]^ This reliance may be because the majority of respondents were predominantly educated individuals with good access to the internet and/or concerned health care workers. Similar to a previous study conducted in Syria by Shibani et al^[17]^ that showed 37% of participants were willing to receive the vaccination, and 24% would receive the vaccine only under government rules, indicating that vaccine mandates could effectively enhance vaccination rates. This was also demonstrated in several countries around the world which have taken similar measures to combat the pandemic.^[24,25]^ Compared to neighboring nations such as Lebanon, Jordan, and Palestine, our study found that the vaccination rate is significantly lower in Syria.^[26–28]^ However, Syria has been suffering from internal military conflicts since 2011, resulting in widespread casualties, injuries, population displacements, and the collapse of healthcare infrastructure. The emergence of the COVID-19 pandemic has exacerbated the situation, placing the country residents in a uniquely challenging position, dealing with the ramifications of the pandemic and the ongoing warfare. This circumstance is particularly noteworthy given Syria underfunded and ill-equipped healthcare system, which lacks the capacity to effectively manage such a dual crisis.^[29]^ Hesitancy to vaccines in our study could be explained by 2 possible causes; First, a rise in spreading conspiracy theories. Second, the absence of solid and effective government vaccination campaigns and initiatives may be, at least, partially due to long-lasting war.

Reasons for unwillingness to be vaccinated, shown in Figure 1, varied among participants with the primary deterrent being “Fear of the potential side effects.” This reason dominated nearly all related investigations.^[30,31]^ In this context, social media platforms could play a notable role in promoting cognitive biases where people tend to have a negative attitude toward information on the disease itself and its vaccines. People also tend to share painful personal experiences and emotions rather than positive ones, which could have a negative impact, particularly in the context of global health crises like pandemics.^[32,33]^

Apparently, a significant relationship between vaccination willingness or acceptance, and the perceived benefits and knowledge about the vaccine was observed. This knowledge endorsed subjects like immunity, side effects awareness, and community/self-protection. The vaccinated group had a high knowledge rate and relied on credible sources for information. This is especially important because the majority of the negative beliefs were based on misinformation or conspiracy theories.^[32]^ Our findings, however, revealed a lack of significant correlation between vaccination status and using social media platforms as a main source of information (*P* = .59) but, several published researches have shown that social media could facilitate the diffusion of anti-vaccine beliefs and conspiracy theories. For instance, a previous study that took place in the United States found an increase in the “infertility” search volume after the rollout of the COVID-19 vaccine.^[34]^ This can be explained by the vast popularity of social media as a source of information that is more accessible, rapid, and sometimes less politically oriented than other options. On the other hand, good use of social media allows authority stakeholders to educate the hesitant to accept the vaccine and promote COVID-19 vaccinations in a reasoned and orderly manner.^[6,20,35]^ In our study, Interestingly, highly trusting social media information showed a significant association with high rates of vaccine acceptance. In the same regard, 2 other main variables had a similar significant association with vaccine acceptance, which were working in a health-related field or having good knowledge about vaccines. Meanwhile, being female, a city resident, or having a previous infection had a negative impact on vaccination decisions.

Conspiracy theories were significant factors in our study. 33% stated that this variant was the main information category posted on social media that influenced their vaccination decision. This highlights the participants’ awareness of the infodemic of misinformation accompanying the COVID-19 vaccine. In response to that, some health organizations, like WHO, have collaborated with some technology and social media companies to tackle this misinformation and limit the related harmful content circulation.^[36]^

Despite having a low vaccination rate, the participants in our study were rather aware of the risks of misusing and trusting public, potentially inaccurate, information. Therefore, they sought to verify its accuracy, and the majority agreed on increasing monitoring measures as it could influence public opinion toward specific decisions. Compared to users of other social media platforms, X and LinkedIn users had greater vaccination rates. However, in another study, respondents who were willing to get vaccinated did not trust what has been spreading neither on Facebook nor on X.^[37]^ Relating to that, the CDC recommends a list of tools to address and collect opinions shared via social media in order to implement effective procedures to overcome the misunderstanding.^[38]^

The intersection of continuous armed conflict in Syria, weakened healthcare infrastructure, and pervasive misinformation presents a multifaceted challenge for public health authorities. The protracted conflict has severely disrupted healthcare services, making it difficult to implement comprehensive vaccination campaigns.^[39]^ The spread of misinformation and certain conspiracy theories detailed in our findings on social media is particularly detrimental in a country where access to reliable information is already compromised. This environment not only fosters vaccine hesitancy but also undermines trust in health authorities and international health organizations. Addressing these issues requires tailored public health strategies that consider the socio-political nuances of Syria. For example, leveraging community leaders and local influencers who are trusted by the population could be more effective than traditional media campaigns. Furthermore, efforts for enhancing internet infrastructure in rural areas could mitigate the digital divide that hampers effective communication and education about the vaccine. These approaches could help counteract the spread of misinformation and improve vaccine uptake in a context as complex as Syria.

## 5 Limitations

Our research has a number of limitations that need to be considered. No causal relationship between the variables and the outcome can be concluded because this was an observational study carried out at a single moment in time. Secondly, as an online-based study primarily distributed via social networks, certain age groups, particularly the elderly, were not appropriately targeted due to their limited engagement with these platforms. Additionally, internet interruptions in many rural areas of Syria further limited the generalizability of our results. On the other hand, the majority of our respondents had college degrees and worked in the healthcare field. These groups typically have a higher interest in and enthusiasm for participating in scientific research aimed at raising local health awareness and enhancing health policies. This may have led to an overrepresentation of educated individuals who were more informed about the COVID-19 pandemic and the importance of vaccination compared to the general public. Lastly, the majority of participants were young and without concomitant diseases, which may have reduced their desire to get vaccinated.

## 6. Conclusion

In the present study, and beyond the question of vaccination, our findings highlight the importance of social media information in health-related decisions in some countries. While the results of this study should be considered in light of its limitations, they emphasize the importance of effective health campaigns, particularly in war-torn areas with limited access to information. Finally, further investigations need to be carried out thoroughly in order to confirm the causality between social media and health decisions and to inform the development of evidence-based health information policy.

## Author contributions

**Conceptualization:** Areej Kahwaji, Thurya Alaryan, Hani Alhelwani, Tamim Alsuliman.

**Data curation:** Areej Kahwaji, Thurya Alaryan, Hani Alhelwani, Moath Salem.

**Formal analysis:** Thurya Alaryan, Tamim Alsuliman.

**Methodology:** Areej Kahwaji, Thurya Alaryan, Tamim Alsuliman.

**Project administration:** Tamim Alsuliman.

**Resources:** Areej Kahwaji, Hani Alhelwani, Moath Salem.

**Supervision:** Thurya Alaryan, Tamim Alsuliman.

**Visualization:** Thurya Alaryan.

**Writing – original draft:** Areej Kahwaji, Tamim Alsuliman.

**Writing – review & editing:** Areej Kahwaji, Tamim Alsuliman.

## Supplementary Material
