# Supplementary material for: Understanding the influence of social media on COVID-19 vaccine acceptance in a war-torn Syria: A cross-sectional study
Source: Medicine (Baltimore). 2024 Aug 9;103(32):e38956. doi: 10.1097/MD.0000000000038956 (PMC11315535; doi:10.1097/MD.0000000000038956)
Supplement: Supplementary file 1 [file medi-103-e38956-s001.docx]

**The impact of social media on COVID-19 vaccine acceptance in a war-torn country: A community-based cross-sectional study**

**Section I**

Dear participants,

This study aims to assess the extent to which individuals living in Syria are aware of COVID-19 vaccinations and their reliance on social media platforms to learn about the vaccine, as well as investigate the impact of spreading false information on people’s refraining from getting vaccinated.

Note that:

1. Participants' answers will be used for the purpose of scientific research only.

2. Participants will not be required to disclose their name, date of birth, or any other personal information.

3. Participants can opt-out at any time while filling out the questionnaire.

4. Since we do not collect the participants' personal data, your answers will be unrecognizable and therefore cannot be eliminated after pressing the "submit" button.

5. For inquiries, you can contact the following email: [res.cov22@gmail.com](mailto:res.cov22@gmail.com)

**Consent form:**

Do you agree to participate in the research “The impact of social media on COVID-19 vaccine acceptance in Syrian community members: A community-based cross-sectional study”?

- Yes
- No

**Section II**

**Participants’ Characteristics**

| 01- Gender | - Male - Female - Rather not say |
| --- | --- |
| 02- Age Group | - 18 - 25 - 26 - 40 - 41 - 60 - Older than 60 |
| 03- Where do you live? | - Northern governorates (Aleppo & Idlib) - Eastern governorates (Deir ez-Zor, Al-Hasakah, Ar-Raqqah) - Central governorates (Homs, Hama) - Western governorates (Latakia, Tartous) - Southern governorates (Damascus, Rif Dimashq, Daraa, As-Suwayda, Quneitra) |
| 04- Marital status | - Single - Married - Divorced / Widowed |
| 05- Working in healthcare-related field | - Yes - No |
| 06- Educational level | - No formal education - Middle school - Highschool - University student - Bachelor’s degree - Post-graduate degree |
| 07- Area of residency | - City - Suburb - Village |
| 08- Financial status | - Basics of living needs only. - Basics of living needs along with complementary expenditure. |

**Section III**

**Beliefs and knowledge about COVID-19 vaccines and willingness to get vaccinated**

| 01- Have you received the vaccine? | - Partially vaccinated - Fully vaccinated - Single-dose vaccine - Didn’t receive the vaccine |
| --- | --- |
| 02- If you have not yet received the vaccine, would you receive the vaccine, if available? | - Yes - No - Only if one of my acquaintances takes it (relatives, friends, etc) - Would take it under compulsory rules only |
| 03- Have you been infected with SARS-CoV-2 before? | - Yes - No |
| 04- How many times have you been infected with the virus? | - Once - Twice - Three times - More than three times - Haven’t been infected before |
| 05- If you were infected with the virus, how was the infection diagnosed? (check all answers that apply) | - PCR test was performed with a nasal or oropharyngeal swab. - I did a Rapid Home Antigen Screening Test. - An x-ray of the lungs was performed, which showed that there are leaky fluids associated with clinical symptoms - Clinical symptoms of the virus appeared (such as shortness of breath, headache, body pain, loss of smell and taste, etc) - Doctor's diagnosis - Telehealth (Diagnosis of a doctor over the phone) |
| 06- Did you develop symptoms of infection after receiving the vaccine? | - Yes - No |
| 07- If you were infected with the virus before, how would you describe the severity of the symptoms? | - Asymptomatic or with minor symptoms only - Moderate symptoms - Severe symptoms - Critical symptoms required a hospital admission |
| 08- Do you suffer from comorbidities? | - Yes - No |
| 09- If you have comorbidities, what are they? (check all answers that apply) | - Type one or two diabetes - Chronic lung diseases - Cancer - Previous or current cardiovascular disease - Chronic kidney disease - Chronic liver disease - Autoimmune diseases - Overweight or obesity - Sickle cell anemia or Thalassemia - I don't have any comorbidities - Other |
| 10- Do you take immunosuppressive drugs? | - Yes - No |
| 11- If you are taking immunosuppressive medications, specify them. (check all answers that apply) | - Corticosteroid drug - Methotrexate - Tacrolimus - Cyclosporin - Azathioprine - I do not take immunosuppressive drugs - Other |
| 12- Do you smoke (including cigarettes, waterpipes, pipes, etc.)? | - Former smoker - Current smoker - Non-smoker |
| 13- If you were vaccinated, what was the main reason for your vaccination? | - limiting the spread of the virus - Getting immunized against the virus and preventing future infections - Fear of complications associated with the virus infection - Previously infected with severe symptoms - Some of my acquaintances died after contracting the virus or suffered from severe complications - The doctor advised me to take the vaccine - Fear of transmitting the virus to children or the elderly at home - the vaccine facilitates travel procedures between countries - I was obligated to take the vaccine, but I didn't take it voluntarily - Other |
| 14- If you have not yet received the vaccine, explain why. | - I am worried about the side effects of the vaccine that may harm the body - The vaccine does not protect against all strains of the virus - Contracting the virus replaces taking the vaccine - Other |
| 15- If you have not yet received the vaccine, which one would you like to get? | - Whatever vaccine is available at the vaccination center - Vaccine recommended by the doctor - The vaccine recommended by one of my acquaintances from outside the medical community - Recommended vaccine on social media |
| 16- Can the COVID-19 vaccine be taken by intramuscular injection? | - Yes - No - I don’t know |
| 17- Do you think the COVID-19 vaccine contributes to | - Completely protecting the recipient from infection - Reducing the severity of the infection or the hospitalization period (time in hospital) - Protect the recipient against some strains only - It does not provide any protection against COVID-19 - I don’t know |
| 18- Do you think that the benefits of the vaccine outweigh the side effects caused by it? | - Yes - No - I don’t know |
| 19- Do you think that vaccination helps protect people who have not received the vaccine from infection? | - Yes - No - I don’t know |
| 20- Do you agree that the vaccine development process goes through three phases of clinical trials, is conducted on thousands of people, and the results are evaluated by health authorities to ensure efficacy and safety? | - Yes - No - I don’t know |

| 21- Do you think that the precautionary measures can be stopped after receiving the vaccine? | - Yes - No - I don’t know |
| --- | --- |
| 22- What are the precautionary measures that must be continued after receiving the vaccine? (check all answers that apply) | - Frequently hand-washing - Mask wearing - Keep a distance of two meters between people - Cover mouth and nose when coughing and sneezing - Avoid going to crowded places - Avoid interacting with people suspected of being infected with the virus - Frequent cleaning of surfaces and objects - Other |
| 23- Do you think that infection with the virus replaces taking the vaccine? | - Yes - No - I don’t know |
| 24- Do you think that the absence of side effects after receiving the vaccine means that the vaccine is not effective? | - Yes - No - I don’t know |
| 25- Do you think the side effects of the vaccine differ between (check all answers that apply) | - Males and females - Age groups - Healthy individuals and patients - Doesn’t differ between the aforementioned categories - I don't know |
| 26- Do you think that side effects after receiving the vaccine mean the person has become contagious? | - Yes - No - I don’t know |
| 27- Is it necessary to fast before receiving the vaccine or to eat certain foods after receiving it? | - Yes - No - I don’t know |

**Section V**

**Social Media frequency use and its effects**

| 01- How many hours per day do you spend on social media? | - 1-2 hours - 2-4 hours - More than 4 hours |
| --- | --- |
| 02- What sources do you use the most to get information about the COVID-19 vaccine? | - Social media platforms - Newscasts - Health care providers (e.g.:doctors) - Health organizations websites (e.g.: Syrian Ministry of Health website) - Published papers in peer-reviewed journals |
| 03- What social media platforms do you use to get information about the COVID-19 vaccine? (check all answers that apply) | - Facebook - WhatsApp - Telegram - Youtube - Instagram - Twitter - LinkedIn - Viber - Other |
| 04- Have you posted/shared information regarding COVID-19 vaccines on social media before? | - Yes - No |
| 05- To what extent do you rely on social media to obtain information about COVID-19 vaccines? | - Highly - Moderately - I don’t rely on it |
| 06- Which of the following information about the vaccine have you obtained through social media? (check all answers that apply) | - The components of the vaccine and its mechanism of action - Administration method - Side effects - Vaccine contraindications - Doses administered - None of the above |
| 07- To what extent do you trust the scientific information on COVID-19 vaccines posted on social media? | - Highly - Moderately - I don’t trust |
| 08- Do you make sure of the credibility of the vaccine information when browsing social media? | - Yes - No - Sometimes |
| 09- Did the information published on the importance of vaccination on social media encourage you to take the vaccine? | - Yes - No |

**Control measurements opinions**

| Items | Strongly agree | Agree | Neutral | Disagree | Strongly disagree |
| --- | --- | --- | --- | --- | --- |
| 10- Do you think that publishing more information about the importance of taking the vaccine on social media encourages people to take the vaccine? |  |  |  |  |  |
| 11- Do you think that information about COVID-19 vaccines posted on Arabic-speaking pages and groups on social media was positively influential? |  |  |  |  |  |
| 12- Do you think that social media has an impact on people's tendency to take a particular vaccine? |  |  |  |  |  |
| 13-Do you think that the measures taken to control information published about medical products such as drugs and vaccines for COVID-19 on social media were sufficient? |  |  |  |  |  |
| 14-Do you believe there should be more controls and oversight of medical information posted on social media, such as COVID-19 drugs and vaccines? |  |  |  |  |  |

| 15-in your opinion, what is the most common category of information published on social media that contributes to people's reluctance to take the vaccine? | - Vaccine side effects - Information questioning the credibility of the product source - Information promoting the ineffectiveness of the vaccine in protecting against infection or reducing the severity of symptoms in the event of infection - Information questioning the credibility of the health institution providing the vaccine - Conspiracy theories - Other |
| --- | --- |
| 16- What are the most conspiracy theories about COVID-19 vaccines you've read on social media? | - The implications of the virus are exaggerated by the media and do not require a vaccine. - The vaccine causes irreversible genetic defects. - Vaccines deliberately control and harm vaccinators by implanting slices in their bodies. - The vaccine contains tissue from aborted fetuses. - The pandemic is part of the pharmaceutical manufacturing companies' deceiving scheme in order to increase their profits after imposing vaccination. - Vaccines cause death after several years after receiving. |
| 17- In your opinion, what is the best way to encourage people to receive the vaccine in the event of future pandemics? | - implement public awareness campaigns. - Disseminating vaccination programs widely, including advertisements on social media and television. - Establishment of mobile vaccination centers. - Sharing vaccination reels and videos of public figures and celebrities. |

|  |
| --- |
